# Evaluation of the antimicrobial management of intracranial suppurative infections in a single pediatric institution

**DOI:** 10.1017/ash.2025.10105

**Published:** 2025-08-15

**Authors:** Cameron E. Bizal, Alaina N. Burns, Rana E. El Feghaly, Brian R. Lee, Ann L. Wirtz

**Affiliations:** 1 Department of Pharmacy, Children’s Hospital Colorado, Aurora, CO, USA; 2 Children’s Mercy Kansas City, Kansas City, MO, USA; 3 Department of Pediatrics, Children’s Mercy Kansas City, Kansas City, MO, USA; 4 University of Missouri-Kansas School of Medicine, Kansas City, MO, USA

## Abstract

**Objective:**

To describe the antimicrobial management of and examine the etiology of intracranial suppurative infections (ISIs) at a single pediatric institution.

**Design:**

Retrospective review.

**Patients:**

We included children hospitalized at a 367-bed freestanding pediatric institution for treatment of an ISI (epidural or subdural empyema, brain abscess) between January 1, 2015, and September 30, 2023. ISIs were identified using international classification of diseases 9/10 discharge diagnosis codes.

**Methods:**

We collected data regarding patient characteristics, infection etiology and complications, antimicrobial choice and route (empiric, definitive, and outpatient), microbiology results, treatment duration, and treatment-related outcomes from the electronic health record.

**Results:**

A total of 72 patients met inclusion criteria. Most patients received a third- or fourth-generation cephalosporin, metronidazole, and vancomycin empirically (69.4%), while a third- or fourth-generation cephalosporin in combination with metronidazole was the most common definitive regimen (63.9%). Almost half of patients (44%) were transitioned to an entirely oral antibiotic regimen, after a median of 27 days of intravenous therapy. The median duration of antimicrobial therapy was 45 days (interquartile range = 33,56). Organisms in the *Streptococcus anginosus* group were the most common pathogens identified (62.5%). Treatment-related complications occurred in 12 (16.7%) patients.

**Conclusions:**

Empiric therapy targeting resistant gram-positive organisms was not required to treat ISIs at our institution. Further data are needed on timing and requirements for oral antibiotic transition and treatment duration. In the future, there is opportunity for multi-institutional collaboration and data-sharing to determine the most appropriate management of pediatric ISIs.

## Introduction

Intracranial suppurative infections (ISIs), such as brain abscesses, subdural empyemas, and epidural empyemas, are challenging infections to treat as there is limited evidence to guide antimicrobial therapy, especially in children.^1^ The most common pathogens recovered from these infections include viridans group streptococci (VGS) and *Staphylococcus aureus*, but gram-negative aerobic pathogens and anaerobes may also be present.^2–5^ Historically, these infections occurred infrequently in pediatric patients; however, there was a notable increase in ISIs following the COVID-19 pandemic.^6,7^

Treatment of pediatric ISIs requires empiric use of broad-spectrum antibiotics, with most guidance derived from retrospective studies, case reports in adults, or expert opinion.^3–5,8^ Recent single-center studies report that majority of patients receive vancomycin, a third-generation cephalosporin, and metronidazole empirically.^3–5^ In contrast, published guidelines from the European Society of Clinical Microbiology and Infectious Diseases (ESCMID) for the treatment of brain abscesses recommend only a third-generation cephalosporin and metronidazole for immunocompetent patients.^8^ In the United States, vancomycin or linezolid are frequently used adjunctively for ISI treatment given the concern for methicillin-resistant *Staphylococcus aureus* (MRSA) or penicillin- and cephalosporin-resistant *Streptococcus* spp.^9–12^

There are several other unanswered questions surrounding the treatment of ISIs in children such as feasibility of transition to an all-oral antibiotic regimen. Given concern of adequate penetration of oral antibiotics into the central nervous system (CNS), the proportion of patients transitioned to a fully oral regimen is variable in the literature.^3–5,13–16^ Duration of treatment is also variable.^3–7^ The ESCMID guidelines recommend 6–8 weeks of treatment, conditionally.^8^

Given the unanswered questions and clinical controversies, the primary objective of this study was to describe the antimicrobial management of ISIs at a single institution over an eight-year period. A secondary objective was to examine the etiology of ISIs.

## Methods

### Design, setting, and population

We performed a single center, retrospective review of patients being treated for an ISI (brain abscess, subdural empyema, or epidural empyema) between January 1, 2015, and September 30, 2023. Children’s Mercy Kansas City is a 367-bed, free-standing, pediatric, academic medical center located in the Midwest, which serves a five state, 100-county region.

Our institution implemented a clinical pathway for ISI management in May 2020. The pathway recommends empiric intravenous (IV) ceftriaxone and metronidazole, with or without vancomycin for a minimum of 2 weeks before potential transition to oral therapy if significant clinical improvement and a highly bioavailable antibiotic is an option. Vancomycin is utilized per provider preference but is prescribed often in patients with a MRSA history or a severe clinical presentation. It recommends 4–8 weeks of antibiotic therapy based on surgical interventions, clinical response, and team judgment.

Patients were included if they were between 3 months and 23 years of age with an encounter International Classification of Diseases (ICD)-9 code of 324.0 (intracranial abscess) or 324.9 (intracranial and intraspinal abscess of unspecified site) or ICD-10 code of G06.0 (intracranial and intraspinal abscess and granuloma) or G06.2 (extradural and subdural abscess, unspecified) as primary or secondary diagnosis. We excluded patients with a spinal epidural abscess; with ISI caused by a parasite, fungus, or mycobacterium; who were immunocompromised; who had undergone a neurosurgical, otolaryngological, or facial surgery within 90 days of presentation; who had a CNS device prior to presentation; who were admitted to the neonatal intensive care unit; and patients who were managed at an outside hospital for more than 24 hours prior to presentation.

### Study outcomes

Patient characteristics included age, sex, race, reported historical antimicrobial adverse reactions, year of admission, length of hospitalization, and need for intensive care unit (ICU) admission. Presence of complex chronic conditions was captured using the Complex Chronic Condition classification system.^17^ Infection characteristics including type of ISI, presumed originating infection, complications on initial imaging, and number of intracranial (eg, craniotomy) and non-intracranial (eg, mastoidectomy) surgical interventions were collected. If patients had more than one type of ISI, they were included within the category of the more invasive ISI for analysis (ie, a patient with an epidural and subdural abscess would be included only in the subdural category). Antibiotic treatment regimens, including antibiotic choice and route, were included for three different timepoints: empiric, definitive, and outpatient regimens. Empiric regimens were considered the first antibiotic agents started within 24 hours after the diagnosis of ISI was made and prior to any culture results. Preoperative prophylactic antibiotics were disregarded. For standardization across all patients, we defined definitive regimens as antibiotics each patient received on the last day of hospitalization, excluding single doses of oral antibiotics initiated as a trial. Outpatient regimens included any antibiotics continued beyond the inpatient encounter. Data on transition to an entirely oral antibiotic regimen, including the proportion of patients transitioned, antibiotics utilized, and timing of transition, was collected. Patients receiving oral metronidazole while still receiving another IV agent were not included in the group of patients transitioned to an oral regimen. Total duration of treatment was calculated using the difference between day of initial empiric antibiotic and day of final antibiotic dose received. Additionally, the duration of treatment with antibiotics targeting resistant gram-positives (eg, MRSA) was collected for each patient. These antibiotics included ceftaroline, clindamycin, dalbavancin, daptomycin, doxycycline, linezolid, minocycline, oritavancin, sulfamethoxazole/trimethoprim, tedizolid, telavancin, and vancomycin. We recorded any treatment-related complications including treatment failure, defined as re-initiation of antibiotics within 90 days of discharge for the same diagnosis; mortality; and re-admission within 90 days for the same diagnosis or a complication thereof for all patients.

A secondary objective was to evaluate the etiology of ISIs. Our in-house microbiology laboratory follows guidance published by the American Society for Microbiology Clinical Microbiology Procedures Handbook.^18^ We collected microbial culture information for each patient with any positive intraoperative intracranial, intraoperative sinus or adjacent, lumbar puncture, and/or blood cultures. All positive culture results were recorded, and each organism was only counted once per patient if it grew on several cultures of the same specimen type. The proportion of patients with aerobic pathogens, polymicrobial infections, and anaerobic pathogens were collected using all patients, even those without positive cultures. We collected duration of antibiotics received prior to culture collection. Susceptibility information, if available, was collected for cultures positive for *Staphylococcus aureus*, VGS, and *Streptococcus pneumoniae*.

### Statistical analysis

We collected study data from a retrospective chart review of the electronic health record (EHR) and incorporated them into REDCap electronic data capture tools hosted at Children’s Mercy Kansas City.^19,20^ Study data were exported into Microsoft® Excel®, with descriptive statistics performed to report patient, infection, and treatment characteristics. This study was approved by the Institutional Review Board at Children’s Mercy Kansas City.

## Results

**Figure 1.**
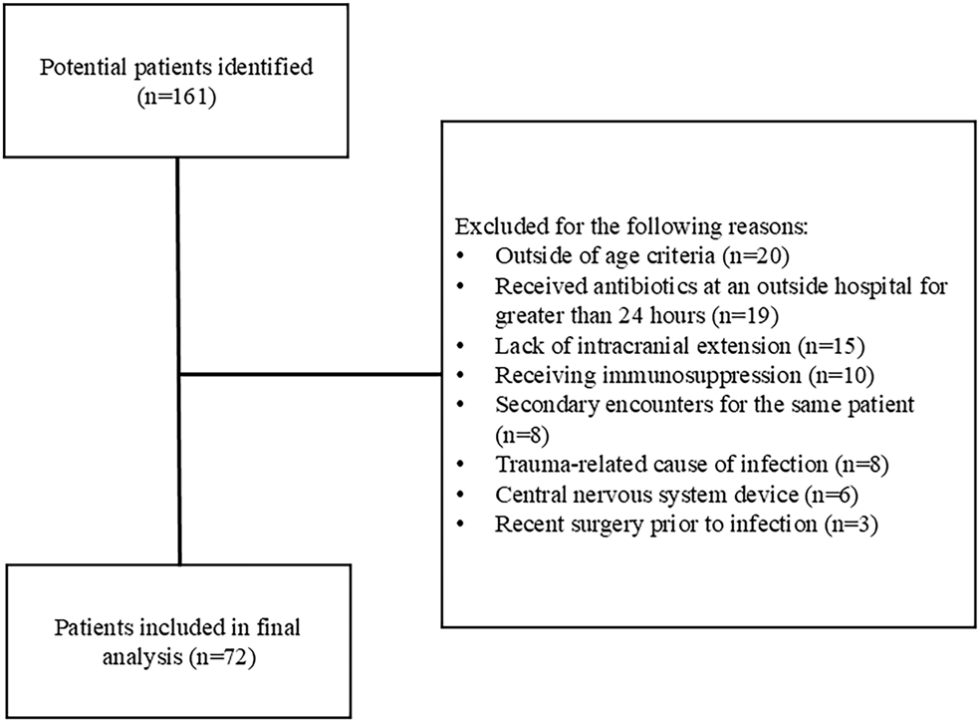
Flow chart of study population.

Between January 1, 2015, and September 30, 2023, we identified 161 patients. Eighty-nine patients met exclusion criteria, leaving 72 unique patients in the final analysis (Figure 1).

Prior to the onset of the COVID-19 pandemic (2015–2019), there was a median of 6 cases per year (interquartile range (IQR): 4,9), and after the onset of the COVID-19 pandemic (2020–2023), there was a median of 11 cases per year (IQR: 4,17). Most patients had a subdural (41.7%) or epidural empyema (33.3%), whereas brain abscesses were less common (25%) (Table 1). Compared to other groups, patients with brain abscess more commonly had an unknown source of infection (27.8%) or infection from hematogenous spread (11.1%) (Table 2). Fifty-six (77.8%) patients had an intracranial surgery with a median of 1 surgery (IQR: 1,2). Additionally, 56 (77.8%) underwent a non-intracranial surgery with median of 1 surgery (IQR: 1,2). In total, 44 patients (61.1%) had both intracranial and non-intracranial surgeries. A complication on initial imaging was present in 54.2% of patients.

**Table 1.** Patient characteristics

Characteristic	All patients (n = 72)	Subdural abscess (n = 30)	Epidural empyema (n = 24)	Brain abscess (n = 18)
Median age, in years (interquartile range)	10.5 (6,13)	11 (5, 12.3)	7.5 (3.8, 11.8)	12.5 (10, 14.3)
Female sex, n (%)	24 (33.3)	7 (23.3)	9 (37.5)	8 (42.1)
Race, n (%)				
White	49 (68.1)	20 (66.7)	16 (66.7)	13 (72.2)
Black or African American	10 (13.9)	7 (23.3)	2 (8.3)	1 (5.6)
Hispanic/Latino	5 (6.9)	1 (3.3)	2 (8.3)	2 (11.1)
Multiracial	3 (4.2)	0 (.0)	3 (12.5)	0 (.0)
Asian	2 (2.8)	1 (3.3)	1 (4.2)	0 (.0)
American Indian or Alaska native	1 (1.4)	1 (3.3)	0 (.0)	0 (.0)
Unknown/Refused	2 (2.8)	0 (.0)	0 (.0)	2 (11.1)
Concomitant chronic condition, n (%)	17 (23.6)	6 (20.0)	4 (16.7)	7 (38.9)
Antibiotic adverse reaction, n (%)	9 (12.5)	4 (13.3)	3 (12.5)	2 (11.1)
Hospitalization details				
Length of hospitalization, days (median, interquartile range)	12 (8–21)	18 (11–23)	9 (6–11)	14 (10–26)

**Table 2.** Infection characteristics

Characteristic	All patients (n = 72)	Subdural abscess (n = 30)	Epidural empyema (n = 24)	Brain abscess (n = 18)
Presumed source of infection, n (%)				
Sinusitis	42 (58.3)	21 (70.0)	13 (54.2)	8 (44.4)
Mastoiditis	16 (22.2)	4 (13.3)	11 (45.8)	1 (5.6)
Otitis media	11 (15.3)	5 (16.7)	5 (20.8)	1 (5.6)
Meningitis	7 (9.7)	5 (16.7)	0 (.0)	2 (11.1)
Hematogenous	5 (6.9)	2 (6.7)	1 (4.2)	2 (11.1)
Unknown	5 (6.9)	0 (.0)	0 (.0)	5 (27.8)
Multiple	14 (19.4)	7 (23.3)	6 (25.0)	1 (5.6)
Complications, n (%)				
Septic venous sinus thrombosis	18 (25.0)	9 (30.0)	8 (33.3)	1 (5.6)
Meningitis/ventriculitis	17 (23.6)	7 (23.3)	8 (33.3)	2 (11.1)
Cranial osteomyelitis	16 (22.2)	6 (20.0)	6 (25.0)	4 (22.2)
Brain herniation	1 (1.4)	1 (3.3)	0 (.0)	0 (.0)
Multiple	11 (15.3)	6 (20.0)	4 (16.7)	1 (5.6)
Intensive care unit admission, n (%)	48 (66.7)	25 (83.3)	7 (29.2)	16 (88.9)
Long-term outcomes, n (%)				
Re-admission within 90 days of discharge	8 (11.1)	3 (10.0)	1 (4.2)	4 (22.2)
Re-treatment within 90 days of discharge	4 (5.6)	3 (10)	0 (.0)	1 (5.6)
Mortality within 90 days of diagnosis	0 (.0)	0 (.0)	0 (.0)	0 (.0)

### Antimicrobial treatment and outcomes

The most common empiric antimicrobial regimen included a third- or fourth-generation cephalosporin, metronidazole, and vancomycin (79.2%) (Table 3). The most common definitive antimicrobial regimen included a third- or fourth-generation cephalosporin with metronidazole (72.2%). Of the patients receiving ceftriaxone monotherapy definitively, 80% had subdural abscesses, and all patients receiving ampicillin/sulbactam had an epidural abscess. Antimicrobials targeting resistant gram-positive bacteria were started empirically in almost all patients (93.1%) but were continued definitively in two patients (2.8%). One patient received vancomycin with cultures positive for *Granulicatella adiacens* and another patient who received linezolid with cultures positive for coagulase-negative staphylococci. Patients received a median of 3 days (IQR: 2,5) of antibiotics targeting resistant gram-positive bacteria. Majority of patients (82%) received a definitive antibiotic regimen with broad-anaerobic coverage (ie, metronidazole, ampicillin/sulbactam, and meropenem).

**Table 3.** Antimicrobial treatment regimens (n = 72 patients)

Empiric antimicrobial therapy regimens	Number of patients (%)
Ceftriaxone + metronidazole + vancomycin	50 (69.4)
Cefepime + metronidazole + vancomycin	7 (9.7)
Ceftriaxone + vancomycin	6 (8.3)
Ampicillin/sulbactam	3 (4.2)
Ceftriaxone + metronidazole	2 (2.8)
Ceftriaxone	2 (2.8)
Other^a^	2 (2.8)

Definitive antimicrobial therapy regimens	Number of patients (%)
Ceftriaxone + metronidazole	46 (63.9)
Ceftriaxone	10 (13.9)
Cefepime + metronidazole	6 (8.3)
Ampicillin/sulbactam	4 (5.6)
Other^b^	6 (8.3)

a Other empiric regimens: ampicillin/sulbactam + ceftriaxone + vancomycin + metronidazole (1); ampicillin/sulbactam + vancomycin + metronidazole (1).

b Other definitive regimens: ampicillin (2); ceftriaxone + metronidazole + linezolid (1); oxacillin + rifampin (1); ampicillin + metronidazole (1); meropenem (1).

Most patients (91.7%) were discharged from the hospital on antimicrobials. The remaining six patients completed treatment while hospitalized. Over one-half of discharged patients (69.7%) received outpatient parenteral antibiotic therapy. The median duration of antimicrobials was 45 days (IQR: 33,56) total and 42 days (IQR: 31,50) since last intracranial surgery for patients who underwent surgery. Treatment-related complications occurred in 12 patients (16.7%) (Table 2).

At any point in treatment, 43.8% of patients were transitioned to an entirely oral regimen (Table 4). Patients received a median of 27 days (IQR: 9,41) of IV antimicrobials prior to oral transition. The majority of patients who transitioned had an epidural empyema (65.6%). Patients transitioning to oral therapy underwent surgical procedures more frequently and had more complications on initial imaging compared to those who were not transitioned (Table 4). Amoxicillin/clavulanate was the most common oral antimicrobial regimen utilized (37.5%). Five patients who transitioned to oral therapy (5/32, 15.6%) and 7 patients who were not transitioned (7/40, 17.5%) experienced treatment-related complications. For patients transitioned to oral therapy, the median duration of IV therapy before oral transition (43 d (IQR:35,47)) was longer in those who experienced treatment-related complications compared to those who did not experience complications (24 d (IQR: 8,35)).

**Table 4.** Characteristics of patients transitioned to entirely oral antimicrobial regimens

Characteristic	Patients switched to oral antibiotics (n = 32)	Patients not switched to oral antibiotics (n = 40)
Oral antimicrobial regimen, n (%)		
Amoxicillin/clavulanate	12 (37.5)	–
Levofloxacin + metronidazole	9 (28.1)	–
Amoxicillin	4 (12.5)	–
Fluoroquinolone monotherapy	4 (12.5)	–
Other^a^	3 (9.4)	
Location of infection, n (%)		
Epidural empyema	17 (53.1)	7 (17.5)
Subdural empyema	10 (31.3)	20 (50.0)
Brain abscess	5 (15.6)	13 (32.5)
Hospitalization details		
Length of hospitalization, days (median, interquartile range)	9 (6,13)	21 (10,28)
Intensive care unit admission, n (%)	19 (59.4)	29 (72.5)
Intracranial surgical procedure, n (%)	26 (81.3)	30 (75)
Non-intracranial surgical procedure, n (%)	31 (96.9)	25 (62.5)
Complication at presentation, n (%)	24 (75.0)	14 (35.0)
Long-term outcomes, n (%)		
Re-admission within 90 days of discharge	4 (12.5)	4 (10.0)
Mortality within 90 days of diagnosis	0 (.0)	0 (.0)
Re-treatment within 90 days of discharge	1 (3.1)	3 (7.5)

a Other oral antimicrobial therapy: linezolid + metronidazole (2), cephalexin + rifampin (1).

### Microbiology

Of the 72 patients, 68 (94.4%) had a positive culture result from any specimen collected. Most cultures were obtained after antibiotic therapy was initiated (84.7%), with most patients (43.1%) receiving greater than 24 hours of antibiotic therapy prior to culture. VGS, specifically organisms in the S*treptococcus anginosus* group, were most common (62.5%) (Table 5). Approximately one-half (54.2%) of patients had a polymicrobial infection. One or more anaerobic organisms were isolated in 20 patients (27.8%). The breakdown of the most common organisms was consistent proportionally when comparing solely intracranial sources to all culture types (Supplemental Table 1).

**Table 5.** Microbiology results (n = 72 patients)^a^

Bacteria	Number of patients (%)
*Streptococcus anginosus* group	45 (62.5)
Anaerobic organisms^b^	38 (52.8)
Coagulase-negative S*taphylococcus* spp.	24 (33.3)
Methicillin-susceptible *Staphylococcus aureus*	11 (15.3)
Other aerobic organisms^c^	10 (13.8)
*Streptococcus pyogenes*	8 (11.1)
*Haemophilus influenzae*	7 (9.7)
*Streptococcus pneumoniae*	4 (5.6)

a See Supplemental Table 1 for further details.

b Includes: *Actinomyces europaeus, Actinomyces odontolyticus, Bacteroides* spp., *Bifidobacterium longum, Cutibacterium* spp., *Dialister* spp., *Fusobacterium* spp., *Gemella morbillorum, Granulicitella adjacens, Micromonas micros, Parviomonas micra, Porphyromonas* spp., *Prevotella* spp., *Veillonella* spp.

c Includes: *Enterococcus faecalis*, diphtheroids, Other viridans group streptococci, *Pseudomonas aeruginosa, Rothia mucilagiosa, Salmonella* spp.

Of the 46 VGS isolates with susceptibility testing performed, all isolates were susceptible to ceftriaxone and vancomycin, and 44 isolates (91.7%) were susceptible to penicillin. All ten *Staphylococcus aureus* isolates were methicillin susceptible. All four *Streptococcus pneumoniae* isolates were susceptible to penicillin, ceftriaxone, and vancomycin.

## Discussion

This retrospective chart analysis describes the antimicrobial management and etiology of ISIs at a single pediatric hospital over an 8-year period. We did not identify any gram-positive bacteria resistant to β-lactam antibiotics, and very few patients were treated definitively with antibiotics targeting resistant gram-positive organisms. Almost one-half of patients were transitioned to an entirely oral antimicrobial regimen, although characteristics and timing were variable. Treatment duration varied, falling between 6 and 8 weeks overall.

The results from this study suggest that, in our area, use of empiric antibiotics covering resistant gram-positive organisms may not be necessary in previously healthy patients with community-acquired ISIs. These data have been replicated in other single-center retrospective reviews which found greater than 90% of isolated gram-positive organisms were susceptible to either third- or fourth-generation cephalosporins.^1,8,10,21^ Rates of intracranial MRSA and cephalosporin-resistant *S. pneumoniae* are decreasing, further limiting the utility of empirically covering for such organisms.^13,22^

Within our study, most patients received only three days of resistant gram-positive antibiotic treatment. Our findings are comparable to other single-center studies in which majority of patients only received vancomycin for a median of five to six days before discontinuation.^3,4^ These data, combined with the ESCMID guideline recommendations, question the need for empiric resistant-gram positive coverage in all patients with ISI.^9^ Unnecessary use of vancomycin can lead to renal toxicity, although less common with short durations, increasing central line entries, unnecessary laboratory monitoring, development of antibiotic resistance, and increased cost.^23–27^ Conversely, the presence of resistant gram-positive organisms in ISIs is not impossible, and failure to provide adequate coverage could lead to poor patient outcomes.^4,27^A single-center study of 54 patients with ISIs identified MRSA in 2 patients and cephalosporin-resistant VGS in 2 patients.^4^ Larger studies are needed to determine whether the benefits of avoiding of vancomycin truly outweigh the risks of inadequate coverage in these patients. Lastly, some data suggest MSSA may be more prevalent than MRSA in ISIs.^13^ MSSA was isolated in cultures of 10 patients within this study. Majority of these patients (60%) received cefepime whereas a small proportion (20%) received ceftriaxone. While ceftriaxone is more narrow-spectrum and has convenient dosing, we speculate providers felt uncomfortable utilizing ceftriaxone for MSSA treatment given clinical controversies around adequate MSSA coverage in severe infections.^28^

Historically, only IV antibiotics were provided as standard of care for ISIs; however, in the early 2000s, experts proposed the transition to oral antimicrobials after 1–2 weeks of IV therapy due to improved adherence, quality of life, decreased complications, and overall reductions in costs.^29,30^ Clinicians are often concerned about the use of oral antibiotics for ISIs given reduced bioavailability for some antibiotics and largely unknown degree of CNS penetration.^31^ Within our study, less than one-half of patients were transitioned to oral antibiotics. Most patients had some degree of source control (eg, surgery) and overall appeared less critical as indicated by shorter length of hospitalization and fewer ICU admissions. Majority of the patients who were transitioned had epidural abscesses, which is likely explained by the fact that CNS penetration of antibiotics may not be necessary when infection occurs outside of the dura.^32^ A recent survey including 49 pediatric institutions from the Sharing Antimicrobial Reports for Pediatric Stewardship (SHARPS) Collaborative confirmed 57% of institutions transition patients with epidural abscesses to entirely oral regimens, mostly amoxicillin/clavulanate or a fluoroquinolone.^16^ Within other pediatric studies, 0–57% of patients with ISI were transitioned to entirely oral therapy, with trends supporting more oral therapy in patients who had mastoiditis, underwent one or fewer surgical procedure, were not admitted to the ICU, or were not bacteremic.^3–5,13–16,33^ Similar to our study, time to oral transition ranged from 7 days to 31.5 days, and of those who received oral therapy, the most common regimens utilized included amoxicillin/clavulanate and levofloxacin combined with metronidazole.^3–5,13–16,33^ All these antibiotics, especially levofloxacin and metronidazole, are all highly bioavailable.

Use of oral antibiotics for ISIs has not been associated with increased risk of short- or long-term neurological sequelae or treatment failure.^8,28,29,31,15,34–36^ Within our study, treatment-related outcomes were numerically similar between patients receiving oral versus intravenous therapy, but negative outcomes were uncommon overall. The ideal timing of IV to oral antibiotic transition is unknown. Larger studies are needed to determine populations who may be eligible for oral transition; however, based on these data, oral therapy could be considered in patients with epidural abscesses, a less severe presentation, or with causative organisms susceptible to highly bioavailable antibiotics such as fluoroquinolones and metronidazole.

We found duration of therapy to be variable. A single-center study of 95 children with ISIs reported an average duration of around 12 weeks.^8^ Conversely a single-center study of 53 children with ISIs reported an average duration of 4 weeks.^22^ Some literature suggests a shorter durations (20–30 d from procedure) may be considered for patients who have undergone drainage of brain abscesses.^36^ Within our study, duration of therapy was approximately 6 weeks, aligning with ESCMID guidelines for management of brain absess.^2,9^ At this time, we do not have strong evidence to support a standardized duration for all patients presenting with ISIs, but instead duration should continue to be based on clinical presentation and treatment response.

As a single center, retrospective, chart review, there are intrinsic limitations. It is possible that patients may have inadvertently been excluded due to lack of the included ICD code. We excluded a large portion of patients due to treatment at an outside hospital for more than 24 hours. Patients underwent high degrees of pretreatment prior to the obtainment of cultures, which may have influenced culture results. Our institutional clinical pathway was developed mid-2020 and may have influenced cases that occurred after development. Additionally, after the increase in cases nationally postCOVID-19 pandemic, our results may primarily reflect more contemporary management. In patients receiving vancomycin, we did not collect vancomycin levels. While we saw low rates of resistant gram-positive organisms, these results are not generalizable to areas with higher rates. As we collected only cultures with a positive result, it is unknown how many total cultures were obtained and whether all patients had both aerobic and anaerobic cultures obtained with each procedure. Finally, with infrequent treatment complications and a limited sample size, this study is unable to address the association between antimicrobial treatment and patient outcomes.

ISIs are treatment dilemmas, especially in pediatric patients. Although antibiotics targeting resistant gram-positive organisms have been included in empiric regimens, these are not necessary at our institution based on culture and treatment data. Despite limited guidance, some patients were transitioned to an entirely oral antimicrobial regimen. This was variable, highlighting the need for further research and established oral transition protocols. The authors are conducting a multi-institutional pediatric study which will assess appropriateness and timing of this transition and the ideal duration of treatment on a larger scale.

## Supporting information

10.1017/ash.2025.10105.sm001 Bizal et al. supplementary material Bizal et al. supplementary material

## Supplementary material

The supplementary material for this article can be found at https://doi.org/10.1017/ash.2025.10105.
