# Supplementary material for: Evaluation of the antimicrobial management of intracranial suppurative infections in a single pediatric institution
Source: Antimicrob Steward Healthc Epidemiol. 2025 Aug 15;5(1):e186. doi: 10.1017/ash.2025.10105 (PMC12394020; doi:10.1017/ash.2025.10105)
Supplement: Bizal et al. supplementary material [file S2732494X25101058sup001.docx]

**Supplemental Table 1.** Microbiology results by culture type (n=72)

| **Intracranial surgical cultures** | **Number of patients** |
| --- | --- |
| Viridans group s*treptococci* | 37 |
| *Streptococcus intermedius* | 30 |
| *Streptococcus constellatus* | 4 |
| *Streptococcus anginosus* | 2 |
| Other viridans group s*treptococci* | 2 |
| Coagulase-negative *Staphylococcus* spp. | 11 |
| *Prevotella* spp. | 9 |
| *Fusobacterium* spp. | 4 |
| Methicillin-susceptible *Staphylococcus aureus* | 4 |
| *Streptococcus pyogenes* | 3 |
| *Cutibacterium* spp. | 2 |
| *Parvimonas micra* | 2 |
| *Streptococcus pneumoniae* | 2 |
| *Actinomyces spp.* | 1 |
| *Bacterioides* spp. | 1 |
| *Dialister pneumosintes* | 1 |
| *Diphtheroids* | 1 |
| *Gemella morbillorum* | 1 |
| *Granulicatella adiacens* | 1 |
| *Haemophilus influenzae* | 1 |
| *Micromonas micros* | 2 |
| *Porphyromonas* spp. | 1 |
| *Pseudomonas aeruginosa* | 1 |
| *Rothia mucilaginosa* | 1 |
| *Veillonella* spp. | 1 |
|  |  |
| **Non-intracranial surgical cultures** | **Number of patients** |
| Viridans group *Streptococci* | 22 |
| *Streptococcus intermedius* | 14 |
| *Streptococcus constellatus* | 4 |
| *Streptococcus anginosus* | 2 |
| Other viridans group s*treptococci* | 2 |
| Coagulase-negative *Staphylococcus* spp. | 10 |
| Methicillin-susceptible *Staphylococcus aureus* | 6 |
| *Streptococcus pyogenes* | 6 |
| *Bacterioides* spp. | 4 |
| *Prevotella* spp. | 4 |
| *Cutibacterium* spp. | 3 |
| *Fusobacterium* spp. | 3 |
| *Actinomyces spp.* | 2 |
| *Diphtheroids* | 2 |
| *Haemophilus influenzae* | 2 |
| *Bifidobacterium longum* | 1 |
| *Enterococcus* spp. | 1 |
| *Pseudomonas aeruginosa* | 1 |
| *Streptococcus pneumoniae* | 1 |
|  |  |
| **Blood cultures** | **Number of patients** |
| *Streptococcus intermedius* | 7 |
| *Haemophilus influenzae* | 3 |
| Coagulase-negative *Staphylococcus* spp. | 2 |
| *Salmonella* spp. | 1 |
| *Streptococcus pneumoniae* | 1 |
| *Streptococcus pyogenes* | 1 |
|  |  |
| **Lumbar puncture cultures** | **Number of patients** |
| *Haemophilus influenzae* | 3 |
| Coagulase-negative *Staphylococcus* spp. | 1 |
| *Salmonella* spp. | 1 |
| *Streptococcus intermedius* | 1 |
